# Changes of dry eye parameters after small incision lenticule extraction surgery in patients with different ocular surface disease index scores

**DOI:** 10.1038/s41598-023-49645-6

**Published:** 2024-01-09

**Authors:** Ge Cui, Tianjiao Wang, Yu Di, Shan Yang, Ying Li, Di Chen

**Affiliations:** 1 Department of Ophthalmology, Peking Union Medical College Hospital, Chinese Academy of Medical Sciences & Peking Union Medical College, Beijing, China; 2 Key Laboratory of Ocular Fundus Diseases, Chinese Academy of Medical Sciences & Peking Union Medical College, Beijing, China

**Keywords:** Medical research, Signs and symptoms

## Abstract

To evaluate the changes of dry eye parameters after small incision lenticule extraction (SMILE) surgery in patients with different ocular surface disease index (OSDI) scores. Prospective research. Participants were divided into two groups: Group A, OSDI < 13; and Group B, OSDI ≥ 13. The OSDI scores, tear meniscus height (TMH), first non-invasive tear film break-up time (NIBUT-First), and meibomian gland loss (MGL, %) were recorded at postoperative 1 -week and 1-month.113 eyes (57 patients) were enrolled, 70 eyes in Group A, and 43 eyes in Group B. In Group A, the OSDI scores significantly increased at 1-week and 1-month postoperative (all P < 0.001); the TMH, NIBUT-First and lipid layer grade significantly decreased at postoperative 1-week (P = 0.003, 0.005, 0.007, 0.004, respectively), but returned to preoperative level at 1-month postoperative. In Group B, only the lipid layer grade significantly decreased at postoperative 1-week (P < 0.05). Patients with different preoperative OSDI scores may experience different changes early after SMILE surgery. Patients with OSDI scores < 13 may experience more dramatic changes in dry eye symptoms which would resolve, while subjective complains could still exists at 1 month after surgery.

## Introduction

Dry eye is one of the major concerns after refractive surgery as it could affect the patients’ visual comfort and overall satisfaction^1^. Refractive surgery was considered as a risk factor of dry eye^2^, and a history of refractive surgery was independently related to a reduction of lipid layer thickness (LLT)^3^, corneal sensitivity^4^, and conjunctival goblet cell density^5^. Structural changes in the meibomian gland (MG) were not observed; however, the reduction of MG function caused by refractive surgery may contribute to chronic tear film dysfunction such as reduction of LLT^3^. Studies showed that corneal sensitivity decreased at 1 week, and 1 and 3 months after refractive surgery and recover at 6^6,7^ or 3 months postoperatively^8^. The tear film break-up time (BUT) decreased at 1 week, and 1 and 3 months after refractive surgery and recovered at 6 months postoperatively^9,10^. The tear meniscus height (TMH), which is positively correlated with the lacrimal secretory rate^11^, decreased at 1 week after refractive surgery^9,10^. Photorefractive keratectomy (PRK) does not section the ciliary nerves, inducing fewer dry eye symptoms in the late postoperative period, compared to laser-assisted in situ keratomileusis (LASIK). Compared to PRK, there is a longer period of sensory denervation leading to the complication of dry eyes in LASIK^12^. A significant reduction in postoperative tear production as well as BUT time was seen with LASIK^13,14^, tear production was more reduced 6 months after LASIK than after PRK^15^. In a study by Lee et al., tear secretion and tear film stability were less at 3 months after LASIK than after PRK^15^.

Patients treated with small incision lenticule extraction (SMILE) surgery have better dry eye parameters^9^ and lesser dry eye symptoms than those that were treated with other refractive surgeries^9^. Previous studies^8,9^ mostly observed SMILE surgery-related dry eye at 1, 3, and 6 months postoperatively; however, they rarely compared the early changes in dry eye symptoms between patients with previously normal and abnormal ocular surface disease index (OSDI) scores after SMILE surgery. In this study, the objective and subjective dry eye parameters of patients with different preoperative OSDI scores at 1 week and 1 month after SMILE surgery were compared and analyzed.

## Results

At baseline, the MGL of the upper and lower eyelids in Group A was significantly lower than that in Group B, while TMH was significantly higher than that in Group B (*P* < 0.05, Table 1). Statistical differences in other parameters were not observed between two groups at baseline (*P* > 0.05, Tables 1, 2).

**Table 1:** Preoperative characteristics.

Parameter	Group A (OSDI < 13) (n = 35, 70 eyes)	Group B (OSDI ≥ 13) (n = 22, 43 eyes)	*P* value
Age, years	28.26 ± 4.75	29.63 ± 4.22	0.123
Gender(M/F)	35(7/28)	22(4/18)	0.663
Total OSDI score	3.7 ± 4.1	26.4 ± 8.2	< 0.001***
Ocular symptom score	1.0 ± 1.4	5.6 ± 2.2	< 0.001***
Vision-related score	0.3 ± 0.9	4.1 ± 3.9	< 0.001***
Environmental score	0.4 ± 0.6	2.5 ± 1.5	< 0.001***
CDVA- logMAR	− 0.029 ± 0.039	− 0.028 ± 0.039	0.928
Sphere, D	− 4.43 ± 1.36	− 4.78 ± 1.42	0.198
Cylinder, D	− 0.89 ± 0.60	− 0.67 ± 0.61	0.070
Spherical equivalent, D	− 4.88 ± 1.42	− 5.12 ± 1.43	0.389
CCT	536.43 ± 22.91	531.44 ± 21.56	0.253
Ks	44.21 ± 1.23	44.57 ± 1.15	0.270
Kf	43.02 ± 1.19	43.52 ± 1.26	0.116
TMH, mm	0.25 ± 0.08	0.21 ± 0.06	0.008**
NIBUT-F, s	9.71 ± 6.57	9.85 ± 6.19	0.907
NIBUT-Ave, s	11.24 ± 5.90	12.32 ± 5.42	0.334
MGL-upper (%)	2.75 ± 3.96	8.06 ± 10.6	0.001**
MGL-lower (%)	6.58 ± 9.86	19.2 ± 20.3	0.037*
Eyelid margin abnormalities-upper	1.38 ± 0.52	1.37 ± 0.58	0.708
Eyelid margin abnormalities-lower	1.12 ± 0.32	1.05 ± 0.22	0.233
Conjunctival congestion score	1.28 ± 0.21	1.27 ± 0.28	0.864
Ciliary congestion score	1.18 ± 0.21	1.12 ± 0.24	0.190

*OSDI* Ocular Surface Disease Index, *CDVA* corrected distance visual acuity, *D* diopter, *CCT* central corneal thickness, *Ks* central corneal radius of the steep meridian, *Kf* central corneal radius of the flat meridian, *TMH* tear meniscus height, *NIBUT-F* the first non-invasive tear film break-up time, *NIBUT-Ave* the average non-invasive tear film break-up time, *MGL* meibomian gland loss (%).

*Statistically significant at P < 0.05, *P < 0.05, **P < 0.01, ***P < 0.001. Continuous variables were expressed as mean ± standard deviation.

**Table 2:** The grade of tear film lipid layer.

	M (P25, P75)		Z	*P* value
	Group A	Group B		
Preoperative	4 (3, 5)	4 (2, 5)	0.177	0.467
Postoperative 1-week	3 (2, 4)	2 (2, 3)	2.687	0.127
Postoperative 1-month	3 (2, 4)	3 (2, 4)	0.380	0.992

Ranking variables were expressed as median and percentile and were analyzed by Wilcoxon test.

*Statistically significant at P < 0.05, *P < 0.05, **P < 0.01, ***P < 0.001.

### Changes in dry eye parameters in patients with normal preoperative OSDI scores

**Figure 1:**
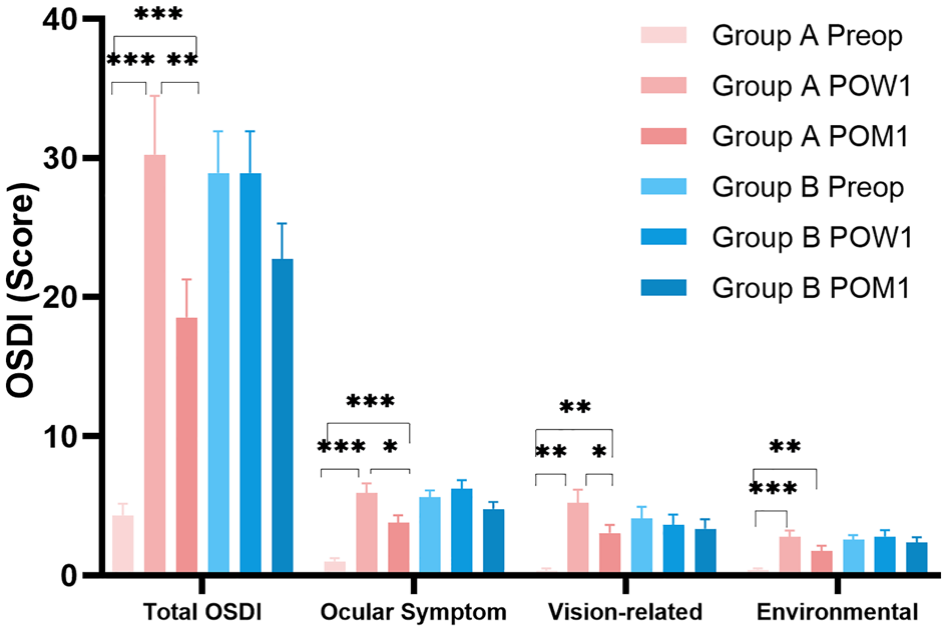
The OSDI scores of two groups at different time points. The total OSDI and three sub-category scores in Group A increased significantly from preoperative to postoperative 1 week postoperatively (all *P* < 0.05), then decreased significantly from postoperative 1 week to postoperative 1 month (all *P* < 0.05). However, the OSDI scores at postoperative 1 month in Group A were still significantly higher than the baseline (all *P* < 0.05). The total OSDI and three sub-category scores in Group B showed no significant differences (all *P* > 0.05). *OSDI* ocular surface disease index.

**Figure 2:**
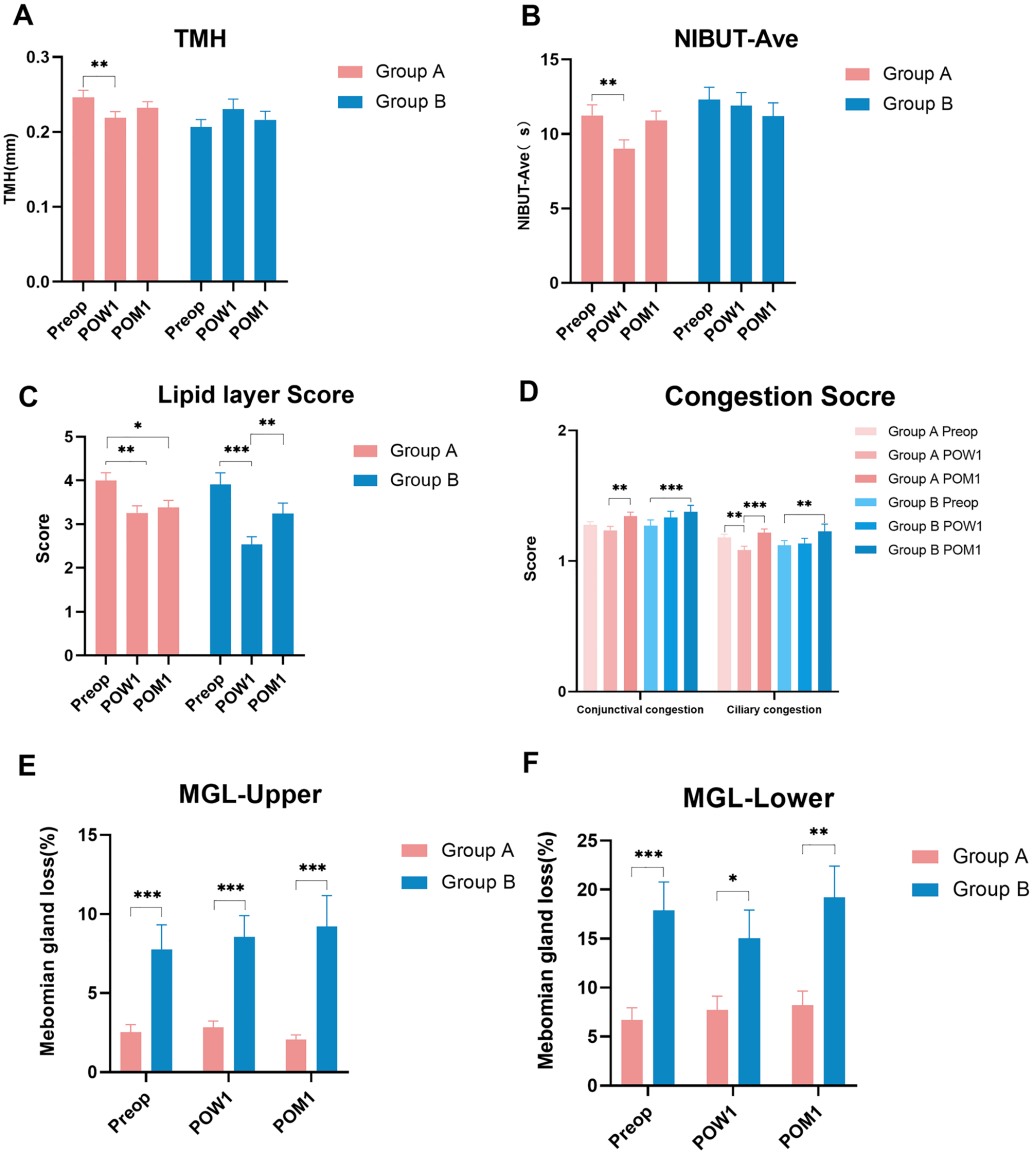
The changes in dry eye parameters in two groups at different time points. (**A**) and (**B)** The TMH and NIBUT-Ave significantly decreased at postoperative 1 week (*P* < 0.01) and returned to preoperative level at postoperative 1 month (*P* > 0.05). (**C**) The grade of tear film lipid layer in Group A at postoperative 1 week and 1 month were both significantly lower than the baseline (*P* < 0.05); the grade of tear film lipid layer in Group B at postoperative 1 week was lower than that at baseline and postoperative 1 month (*P* < 0.01). (**D**) Both the conjunctival and ciliary congestion scores in two groups increased significantly at 1 month postoperatively compared to 1 week postoperatively (all *P* < 0.01). (**E**) and (**F**) The MGL of the upper and lower eyelids in Group A were significantly lower than those in Group B at postoperative 1 week and 1 month (all* P* < 0.05,). TMH = tear meniscus height; NIBUT-Ave = the average non-invasive tear film break-up time; *MGL* meibomian gland loss (%).

The total OSDI score and three sub-category OSDI scores in Group A increased significantly from preoperative to postoperative 1 week (all *P* < 0.05), then decreased significantly from postoperative 1 week to postoperative 1 month (all *P* < 0.05*,* Fig. 1). However, the OSDI scores at postoperative 1 month in Group A were still significantly higher than the baseline (all *P* < 0.05, Fig. 1). The total OSDI and three sub-category scores in Group B showed no significant differences (all *P* > 0.05, Fig. 1). The TMH, NIBUT-First, and NIBUT-Ave significantly decreased at postoperative 1 week (*P* = 0.003, 0.005, 0.007, respectively) and returned to preoperative level at postoperative 1 month (*P* = 0.088, 0.070, 0.595, respectively) (Fig. 2A and B). The grade of tear film lipid layer at postoperative 1 week and 1 month were both significantly lower than the baseline (P = 0.004 & 0.020, respectively) (Fig. 2C**, **Table 2). Both the conjunctival and ciliary congestion scores increased significantly at 1 month postoperatively compared to 1 week postoperatively (P = 0.002 & P < 0.001, respectively) (Fig. 2D). Significant changes in other parameters were not observed at different time points (*P* > 0.05).

### Changes in dry eye parameters in patients with abnormal preoperative OSDI scores

Significant differences in the total OSDI and three sub-category scores were not observed at different time points in Group B (*P* > 0.05, Fig. 1). The grade of tear film lipid layer at postoperative 1 week was lower than that at baseline and postoperative 1 month (all *P* < 0.01, Fig. 2C). Both the conjunctival and ciliary congestion scores increased significantly at postoperative 1 month compared to the baseline (P < 0.001 & P = 0.007, respectively) (Fig. 2D). Significant changes in other parameters were not observed at different time points (*P* > 0.05).

### Comparison of dry eye parameters between two groups after surgery

Significant differences in the total OSDI and three sub-category scores were not observed between two groups at postoperative 1 week and 1 month (*P* > 0.05, Table 3). The NIBUT-Ave and MGL of the upper and lower eyelids in Group A were significantly lower than those in Group B at postoperative 1 week (*P* < 0.05, Fig. 2E and F, Table 3). The MGL of the upper and lower eyelids in Group A were still significantly lower than those in Group B at postoperative 1 month (*P* < 0.05, Fig. 2E and F, Table 3). Statistical differences in other dry eye parameters were not observed between the two groups at postoperative 1 week and 1 month (*P* > 0.05, Table 3).

**Table 3:** Comparison of postoperative OSDI scores and dry eye parameters between Group A and Group B.

Parameter	Postoperative 1-week			Postoperative 1-month		
	Group A (OSDI < 13) (n = 35, 70 eyes)	Group B (OSDI ≥ 13) (n = 22, 43 eyes)	*P* value	Group A OSDI < 13) (n = 35, 70 eyes)	Group B (OSDI ≥ 13) (n = 22, 43 eyes)	*P* value
UDVA-log MAR	− 0.026 ± 0.061	− 0.009 ± 0.101	0.401	− 0.039 ± 0.055	− 0.059 ± 0.086	0.276
Total OSDI score	30.2 ± 25.0	28.9 ± 13.8	0.931	18.5 ± 14.6	22.7 ± 10.5	0.300
Ocular symptom score	5.9 ± 4.2	6.2 ± 2.8	0.642	3.8 ± 2.8	4.8 ± 2.1	0.208
Vision-related score	5.2 ± 5.7	3.6 ± 3.4	0.254	3.0 ± 3.1	3.4 ± 2.7	0.624
Environmental score	2.8 ± 2.7	2.8 ± 2.0	0.925	1.8 ± 2.0	2.4 ± 1.5	0.339
TMH, mm	0.22 ± 0.07	0.22 ± 0.08	0.996	0.23 ± 0.07	0.22 ± 0.07	0.172
NIBUT-First, s	7.09 ± 5.24	9.36 ± 6.54	0.136	8.15 ± 5.98	8.37 ± 6.34	0.552
NIBUT-Ave, s	9.02 ± 4.86	11.90 ± 5.79	0.027*	10.92 ± 5.12	11.19 ± 5.76	0.645
MGL-Upper (%)	3.26 ± 3.49	8.48 ± 9.34	0.035*	2.48 ± 2.40	9.51 ± 13.40	0.016*
MGL-Lower (%)	7.66 ± 11.30	16.20 ± 20.4	0.002**	9.06 ± 11.90	20.60 ± 22.80s	0.006**
Eyelid margin abnormalities-upper	1.57 ± 0.58	1.46 ± 0.60	0.208	1.53 ± 0.53	1.49 ± 0.51	0.826
Eyelid margin abnormalities-lower	1.24 ± 0.46	1.15 ± 0.36	0.197	1.25 ± 0.44	1.15 ± 0.36	0.222
Conjunctival congestion score	1.23 ± 0.26	1.33 ± 0.32	0.267	1.34 ± 0.25	1.38 ± 0.32	0.672
Ciliary congestion score	1.08 ± 0.24	1.13 ± 0.26	0.422	1.22 ± 0.24	1.23 ± 0.36	0.504

*OSDI* Ocular Surface Disease Index, *UDVA* uncorrected distance visual acuity, *TMH* tear meniscus height, *NIBUT-F* the first non-invasive tear film break-up time, *NIBUT-Ave* the average non-invasive tear film break-up time, *MGL* meibomian gland loss (%).

*Statistically significant at P < 0.05, *P < 0.05, **P < 0.01, ***P < 0.001. Continuous variables were expressed as mean ± standard deviation.

## Discussion

In the current study, patients were divided into two groups according to their preoperative OSDI scores; and time-dependent changes in OSDI scores and objective dry eye parameters were observed after SMILE surgery. Patients who underwent SMILE surgery have complaints of dry eye at 1 week postoperatively. However, patients with different preoperative dry eye complaints may experience different changes in dry eye symptoms postoperatively.

The participants in Group A demonstrated a significant increase in OSDI scores at 1 week postoperatively, which was consistent with Li’s study^10^, and the OSDI scores can’t return to preoperative levels at 1 month postoperatively in our study but in Li’s^10^. In our study, although the OSDI scores decreased at 1 month postoperatively, it was still higher than that before surgery. This discrepancy between studies could be explained that we divided patients into Groups A and B. The patients in Group A had more complaints after surgery, while those in Group B thought that the benefits outweigh the discomfort; thus, the trend of the two groups could offset each other. The reversal from low OSDI before surgery to high OSDI after surgery probably because of the reduction of objective dry eye parameters and the adaptation to change in refractive status and visual habit from pre- to post-operative. Specifically, those patients enjoy good subjective visual experience before surgery, and surgical procedures affected their corneal nerves and aberrations, corneal contrast, corneal sensitivity, and visual quality; hence, there was a dramatic contrast of subjective experience after surgery, leading to higher OSDI scores. Furthermore, injury to the corneal sensory nerves after refractive surgery could produce aberrant impulse discharges that might evoke sensations of dryness^16^. For those with high OSDI scores at postoperative 1 week, the increase of OSDI score was transient; thus, clinicians should explain that their condition could improve at 1 month postoperatively. Those patients need more reassurance that their condition will improve in a month or longer.

In terms of objective dry eye parameters in Group A, a transient decrease in TMH, non-invasive tear film break-up time (NIBUT), and grade of lipid layer at postoperative 1 week, and recovery at postoperative 1 month were observed. Possible mechanisms^17–21^ for those changes caused by refractive surgery are as follows: (1) the ablation of the cornea changed the corneal curvature, which in turn decreased tear film stability and distribution, increased evaporation of the tears, and shortened the NIBUT; and (2) the surgical procedures inevitably cut the limbal nerves and affected the corneal microenvironment, leading to decreased neurotrophic influences on the epithelial cells, decreased blinking rate, decreased normal and reflex stimulation of tear production, and eventually reducing the TMH and NIBUT. Patients in Group B experienced lesser dramatic changes in OSDI scores and dry eye parameters at 1 week postoperatively. Their preoperative symptoms are relatively severe; however, the application of eye drops including hormones and artificial tears postoperatively may relieve dry eye symptoms to a certain extent. The MGL in Group A was consistently lower than that in Group B; however, the TMH in Group A was less than that in Group B at 1 postoperative 1 week; thus, it can be speculated that the increase in TMH in Group B was due to some compensatory effect^22^.

The TMH and NIBUT are objective noninvasive quantitative parameters. The height of the lower meniscus may represent the total tear volume better than the upper meniscus^23^; hence, only the lower TMH was analyzed. The TMH in Group A was higher than that in Group B at baseline, whereas the difference disappeared at 1 week postoperatively. The TMH in Group A decreased significantly at postoperative 1 week compared to that at baseline because the lipid layer was thinner then, and the evaporation of the tear film increased correspondingly^24^. The outermost layer of the tear film, the tear film lipid layer, plays an important role in maintaining tear film stability and preventing tear evaporation^25–28^. Tear film lipid layer deterioration leads to tear film instability and evaporative dry eye^29^. In our study, BUT decreased at 1 week, which was consistent with previous studies^9,10^. It appears that TMH, BUT, and the lipid layer were positively correlated with each other^30^, and all those parameters would deteriorate in patients with dry eye. In our study, the TMH, NIBUT, and lipid layer grade in Group A decreased at 1 week postoperatively.

In comparison to other operations, SMILE had better dry eye parameters and substantially fewer subjective complaints^9^. One month after surgery, both SMILE and femtosecond laser-assisted laser in situ keratomileusis (FS-LASIK) showed signs of mild to moderate dry eye condition, however the FS-LASIK group continued to have much more symptoms than the SMILE group after 6 months^1^. The OSDI, BUT, Schirmer, or dry eye score did not differ at 1 month following surgery between FS-LASIK and SMILE, however tear osmolarity was higher in the former^1^. Previous studies discovered that symptoms (OSDI score), signs (BUT)^10^, and tear osmolarity increased more following FS-LASIK than after SMILE at 6 months^1^. OSDI did not differ statistically significantly at any point in time, with the exception of 6 months after surgery (SMILE is lower than FS-LASIK)^10^. The SMILE group had a lower incidence of corneal staining and the SMILE group's mean central corneal sensitivity was higher than the FS-LASIK group's^10^. When compared to the FS-LASIK group 1 and 6 months after surgery, the SMILE group had considerably greater levels of corneal nerve density, long fiber count, and branchings^1^. In addition, SMILE showed increased but decreased inflammatory mediator on the ocular surface with a quicker recovery than FS-LASIK^31^. SMILE involves making a small incision on the anterior stroma, which can lessen the damage to the tear film and corneal nerves, while FS-LASIK causes more damage to the corneal sub-basal nerve^31,32^. At every postoperative time point, however, the central corneal sensation values in the SMILE group were higher than those in the FS-LASIK group. Corneal sensitivity improved in SMILE eyes over FS-LASIK eyes 1 month after surgery before returning to statistically comparable levels at 6 months. Previous studies showed that corneal sensitivity decreased after PRK but rebounded to nearly normal levels after 3 months, these findings could perhaps explain the potential of more rapid corneal re-innervation following PRK and laser-assisted subepithelial keratomileusis (LASEK) than following LASIK^33^. In LASIK, the nerves of the central cornea are cut by the microkeratome, in addition to the laser ablation for myopia correction. Kim et al. reported that lamellar cutting of the cornea during LASIK affects corneal sensitivity, and noted that the cornea did not recover to its pre-operative level even after 6 months, this could explain the greater severity of dry eye disease observed following LASIK compared to other surgeries^34^.

To our best knowledge, this is the first study to compare the postoperative dry eye symptoms of patients with different preoperative OSDI scores after SMILE. Nowadays, SMILE surgery has been increasingly chosen by patients; hence, dry eye after SMILE surgery has been a big challenge for both physicians and patients. However, previous studies rarely compared the early changes in dry eye symptoms between patients with previously normal and abnormal OSDI scores after SMILE surgery. The main limitation of this study was that Schirmer test without anesthesia and corneal fluorescein staining were not performed and the patients were followed for only 1 month. Further researches involving larger sample sizes and longer follow-up times are needed to better describe the association between preoperative OSDI scores and postoperative dry eye.

In conclusion, patients with different preoperative OSDI scores may experience different changes in dry eye symptoms early after SMILE surgery. Patients with low preoperative OSDI scores may have more complaints about dry eye after surgery. There were statistically significant increases in the postoperative OSDI scores in Group A at 1 week compared with preoperative values, and then decreased at 1 month but still higher than preoperative level.

## Materials and methods

### Clinical evaluation

This observational study was conducted at the Department of Ophthalmology, Peking Union Medical College Hospital (PUMCH) from April 2021 to June 2022. The tenets of the Declaration of Helsinki were followed throughout the study and written informed consents were obtained from all patients.

The inclusion criteria were as follows: (1) has − 0.75 to − 8.00 diopters (D) of spherical myopia with astigmatism less than or equal to − 3.00 D, (2) aged ≥ 18 years, (3) has corrected distance visual acuity (CDVA) ≥ 20/20, (4) does not use eye drops or eye medications, and (5) can give informed consent. The exclusion criteria were history of active ocular diseases, systemic conditions, or intake of systemic medications, abnormal binocular vision function, and other contraindications to refractive surgery. G*power 3.1.9.2 software 3 was used to estimate the sample size, and an estimated sample size of 52 was calculated. Finally, a total of 113 eyes of 57 patients were enrolled.

Before surgery, every patient answered the Chinese version of the OSDI questionnaire^35^ that has proven satisfactory diagnostic power. The patients were divided into two groups according to the total OSDI score: Group A, OSDI score < 13 points (normal OSDI scores); and Group B, OSDI score ≥ 13 points (abnormal OSDI scores). Both the total and sub-category scores were recorded and analyzed as previously reported^36^. A comprehensive ophthalmic examination was also performed before surgery, including CDVA, cycloplegic refraction (KR-3500, Topcon, Tokyo, Japan), slit-lamp biomicroscopy, corneal topography (Tomey TMS-4; Tomey, Nagoya, Japan), central corneal thickness (CCT, AL-3000; Tomey, Nagoya, Japan), and dilated fundus examination. DED-1L dry eye analyzer (Chongqing KanghuaRuiming Technology Co., LTD) was applied to obtain the following parameters: (1) TMH, (2) first non-invasive tear film break-up time (NIBUT-F) and average non-invasive tear film break-up time (NIBUT-Ave), (3) grade of lipid layer, (4) meibomian gland loss (MGL, %), and (5) grade of lid margin and meibomian gland orifices. The lipid layer was graded to 7 levels according to the color, distribution and flow of the tear film lipid layer during blinking as previously reported^37^. The MGL was scored quantitatively as previously reported^38,39^. The ciliary and conjunctival congestion were automatically measured and scored according to the redness degree with the Efron Scale (0 = normal, 1 = trace, 2 = mild, 3 = moderate and 4 = severe) as previously reported^37,40^. The eyelid margin abnormalities were evaluated as previously described^41^. The OSDI questionnaire, uncorrected distance visual acuity (UDVA), and DED-1L dry eye analyzer evaluation were performed at 1 week and 1 month postoperatively, respectively.

SMILE surgery was performed by the same experienced surgeon using the VisuMax FS laser (Carl Zeiss Meditec, Jena, Germany). Cap thickness was set at 120 μm with a lenticular diameter of 6.5 mm, incision size of 2 mm, incision position at 120°, and cap diameter of 7.7 mm in all cases. After surgery, tobramycin and dexamethasone eye drops were administrated Qid for the first week and then changed to diclofenac sodium eye drops Qid for three weeks. Routine use of artificial tears (HYCOSAN; EUSANGmbH, Inc) four times a day for at least 3 months and as needed after surgery was prescribed.

### Statistical analysis

GPower 3.1 software^42^ was used to verify if the power of the sample size was enough. Binocular data were enrolled and the relationship between paired measurements from the same participant were evaluated using the intraclass correlation coefficient (ICC) according to a previous study^43^. ICC measurements showed poor-to-fair correlation; thus, data from both eyes of the same participant were included and further adjusted with a linear mixed-effects regression model using the lme4 package in R (version 4.2.1). The statistical differences of the independent variables between groups were determined by two-tailed Student’s t-test and Mann–Whitney test using IBM SPSS Statistics for Windows, version 24 (IBM Corp., Armonk, N.Y., USA); comparisons of the independent variables of the same group at different time points were performed with one-way repeated measures analysis of variance and Bonferroni test using SPSS. Continuous variables that were normally distributed were expressed as mean ± standard deviation, and ranking variables were expressed as median and percentile. For all analyses, a P value of < 0.05 was considered statistically significant. The statistical figures were drawn with GraphPad Prism 9.0 software (GraphPad, San Diego, CA, USA).

### Ethical declarations

The Declaration of Helsinki was followed by all the participant researchers, and the present study protocol was approved by the Institutional Review Board/Ethics Committee of PUMCH. Written informed consent was obtained from each patient before the surgery.

## Data Availability

The data used to support the findings of this study are available from the corresponding author upon request.

## References

[1] Denoyer A, Landman E, Trinh L, Faure JF, Auclin F, Baudouin C (2015). Dry eye disease after refractive surgery: Comparative outcomes of small incision lenticule extraction versus LASIK. Ophthalmology.

[2] The definition and classification of dry eye disease: Report of the Definition and Classification Subcommittee of the International Dry Eye WorkShop (2007). *Ocul Surf*. 2007;5(2):75-92. doi:10.1016/s1542-0124(12)70081-2 10.1016/s1542-0124(12)70081-2 17508116

[3] Jung JW, Park SY, Kim JS, Kim EK, Seo KY, Kim TI (2016). Analysis of factors associated with the tear film lipid layer thickness in normal eyes and patients with dry eye syndrome. Invest. Ophthalmol. Vis. Sci..

[4] Ma KK, Manche EE (2022). Corneal sensitivity and patient-reported dry eye symptoms in a prospective randomized contralateral-eye trial comparing laser in situ keratomileusis and small incision lenticule extraction. Am. J. Ophthalmol..

[5] Albietz JM, Lenton LM (2004). Management of the ocular surface and tear film before, during, and after laser in situ keratomileusis. J. Refract. Surg..

[6] Chung B, Choi M, Lee KY (2020). Comparing dry eye disease after small incision lenticule extraction and laser subepithelial keratomileusis. Cornea.

[7] Demirok A, Ozgurhan EB, Agca A (2013). Corneal sensation after corneal refractive surgery with small incision lenticule extraction. Optom. Vis. Sci..

[8] Wei S, Wang Y (2013). Comparison of corneal sensitivity between FS-LASIK and femtosecond lenticule extraction (ReLEx flex) or small-incision lenticule extraction (ReLEx smile) for myopic eyes. Graefes. Arch. Clin. Exp. Ophthalmol..

[9] Xu Y, Yang Y (2014). Dry eye after small incision lenticule extraction and LASIK for myopia. J. Refract. Surg..

[10] Li M, Zhao J, Shen Y (2013). Comparison of dry eye and corneal sensitivity between small incision lenticule extraction and femtosecond LASIK for myopia. PLoS One.

[11] Holly FJ (1985). Physical chemistry of the normal and disordered tear film. Trans. Ophthalmol. Soc. U. K. (1962).

[12] Sridhar MS, Rao SK, Vajpayee RB, Aasuri MK, Hannush S, Sinha R (2002). Complications of laser-in-situ-keratomileusis. Indian J. Ophthalmol..

[13] Sambhi RS, Sambhi GDS, Mather R, Malvankar-Mehta MS (2020). Dry eye after refractive surgery: A meta-analysis. Can. J. Ophthalmol..

[14] Yahalomi T, Achiron A, Arnon R, Stanescu N, Pikkel J (2023). Dry eye disease following LASIK, PRK, and LASEK: An observational cross-sectional study. J. Clin. Med..

[15] Lee JB, Ryu CH, Kim J, Kim EK, Kim HB (2000). Comparison of tear secretion and tear film instability after photorefractive keratectomy and laser in situ keratomileusis. J. Cataract Refract. Surg..

[16] Belmonte C (2007). Eye dryness sensations after refractive surgery: Impaired tear secretion or "phantom" cornea?. J. Refract. Surg..

[17] Ambrósio R, Tervo T, Wilson SE (2008). LASIK-associated dry eye and neurotrophic epitheliopathy: Pathophysiology and strategies for prevention and treatment. J. Refract. Surg..

[18] Shoja MR, Besharati MR (2007). Dry eye after LASIK for myopia: Incidence and risk factors. Eur. J. Ophthalmol..

[19] Salomão MQ, Ambrósio R, Wilson SE (2009). Dry eye associated with laser in situ keratomileusis: Mechanical microkeratome versus femtosecond laser. J. Cataract Refract. Surg..

[20] Turu L, Alexandrescu C, Stana D, Tudosescu R (2012). Dry eye disease after LASIK. J. Med. Life.

[21] Savini G, Barboni P, Zanini M, Tseng SC (2004). Ocular surface changes in laser in situ keratomileusis-induced neurotrophic epitheliopathy. J. Refract. Surg..

[22] Jung JW, Kim JY, Chin HS, Suh YJ, Kim TI, Seo KY (2017). Assessment of meibomian glands and tear film in post-refractive surgery patients. Clin. Exp. Ophthalmol..

[23] Czajkowski G, Kaluzny BJ, Laudencka A, Malukiewicz G, Kaluzny JJ (2012). Tear meniscus measurement by spectral optical coherence tomography. Optom. Vis. Sci..

[24] Nair S, Kaur M, Sah R, Titiyal JS (2022). Impact of taping the upper mask edge on ocular surface stability and dry eye symptoms. Am. J. Ophthalmol..

[25] Bron AJ, Tiffany JM, Gouveia SM, Yokoi N, Voon LW (2004). Functional aspects of the tear film lipid layer. Exp. Eye Res..

[26] Foulks GN (2007). The correlation between the tear film lipid layer and dry eye disease. Surv. Ophthalmol..

[27] Gokul A, Wang MTM, Craig JP (2018). Tear lipid supplement prophylaxis against dry eye in adverse environments. Cont. Lens Anterior Eye.

[28] Foulks GN, Bron AJ (2003). Meibomian gland dysfunction: A clinical scheme for description, diagnosis, classification, and grading. Ocul. Surf..

[29] Yokoi N, Takehisa Y, Kinoshita S (1996). Correlation of tear lipid layer interference patterns with the diagnosis and severity of dry eye. Am. J. Ophthalmol..

[30] Wang J, Palakuru JR, Aquavella JV (2008). Correlations among upper and lower tear menisci, noninvasive tear break-up time, and the Schirmer test. Am. J. Ophthalmol..

[31] Wong AHY, Cheung RKY, Kua WN, Shih KC, Chan TCY, Wan KH (2019). Dry eyes after SMILE. Asia Pac. J. Ophthalmol. (Phila).

[32] Cai WT, Liu QY, Ren CD (2017). Dry eye and corneal sensitivity after small incision lenticule extraction and femtosecond laser-assisted in situ keratomileusis: A meta-analysis. Int. J. Ophthalmol..

[33] Kohlhaas M, Spoerl E, Boehm AG, Pollack K (2006). A correction formula for the real intraocular pressure after LASIK for the correction of myopic astigmatism. J. Refract. Surg..

[34] Montés-Micó R, Charman WN (2001). Intraocular pressure after excimer laser myopic refractive surgery. Ophthalmic Physiol. Opt..

[35] Yang L, Pazo EE, Zhang Q (2022). Treatment of contact lens related dry eye with intense pulsed light. Cont. Lens Anterior Eye.

[36] Gong Q, Li A, Chen L (2022). Evaluation of dry eye after refractive surgery according to preoperative meibomian gland status. Front. Med. (Lausanne).

[37] Sánchez-González MC, Capote-Puente R, García-Romera MC (2022). Dry eye disease and tear film assessment through a novel non-invasive ocular surface analyzer: The OSA protocol. Front. Med. (Lausanne).

[38] Eom Y, Lee JS, Kang SY, Kim HM, Song JS (2013). Correlation between quantitative measurements of tear film lipid layer thickness and meibomian gland loss in patients with obstructive meibomian gland dysfunction and normal controls. Am. J. Ophthalmol..

[39] Pult H, Riede-Pult BH (2012). Non-contact meibography: Keep it simple but effective. Cont. Lens Anterior Eye.

[40] Sánchez-González JM, De-Hita-Cantalejo C, Martínez-Lara C, Sánchez-González MC (2022). Lipid, aqueous and mucin tear film layer stability and permanence within 0.15% liposome crosslinked hyaluronic acid versus 0.15% non-crosslinked hyaluronic acid measured with a novel non-invasive ocular surface analyzer. J. Clin. Med..

[41] Arita R, Itoh K, Maeda S (2009). Proposed diagnostic criteria for obstructive meibomian gland dysfunction. Ophthalmology.

[42] Faul F, Erdfelder E, Lang AG, Buchner A (2007). G*Power 3: A flexible statistical power analysis program for the social, behavioral, and biomedical sciences. Behav. Res. Methods.

[43] Armstrong RA (2013). Statistical guidelines for the analysis of data obtained from one or both eyes. Ophthalmic Physiol. Opt..

